# Diagnostic challenges and treatment breakthroughs in *Malassezia restricta*-induced meningoencephalitis: a real-world analysis of early mNGS applications

**DOI:** 10.3389/fcimb.2025.1613521

**Published:** 2025-07-18

**Authors:** Jian Wang, Lin Tao

**Affiliations:** ^1^ Department of Neurology, Affiliated Aerospace Hospital of Zunyi Medical University, Zunyi, Guizhou, China; ^2^ Disease Prevention and Control Center of Huaxi District, Guiyang, Guizhou, China

**Keywords:** *Malassezia*, *Malassezia restricta*, meningoencephalitis, metagenomic next-generation sequencing, diagnostic challenges and treatment breakthroughs

## Abstract

**Background:**

*Malassezia restricta* is a lipid-dependent yeast species that commonly colonizes human and warm-blooded animal skin as an opportunistic pathogen. Although strongly associated with dermatological conditions like seborrheic dermatitis, central nervous system (CNS) infections caused by *Malassezia restricta* are remarkably rare. These infections typically present with nonspecific clinical features, leading to frequent diagnostic delays and misdiagnosis.

**Methods:**

We performed a retrospective analysis of five definitive cases of *Malassezia restricta*-induced meningoencephalitis diagnosed at our institution. Comprehensive clinical evaluations included disease onset patterns, symptomatology, laboratory findings, neuroimaging features, therapeutic regimens, and patient outcomes.

**Results:**

All patients exhibited acute onset meningoencephalitis, with headache being the most common presentation symptom, and patients presenting with decreased consciousness showed rapid clinical deterioration. Brain magnetic resonance imaging (MRI) exhibits ring enhancement accompanied by ring diffusion restriction, plays a crucial role in early diagnosis. The cerebrospinal fluid (CSF) demonstrated markedly elevated intracranial pressure and a significant decrease in CSF glucose, vital laboratory markers of critical illness. Metagenomic next-generation sequencing (mNGS) of CSF confirmed *Malassezia restricta* infection in all cases, enabling prompt diagnosis. Early combination therapy with intravenous and intrathecal antifungal agents significantly improved survival outcomes.

**Conclusion:**

*Malassezia restricta*-associated meningoencephalitis represents an extremely rare and life-threatening CNS infectious disease. Since nonspecific early symptoms lead to diagnostic challenges, CSF usually shows a significant increase in intracranial pressure, and a significant decrease in CSF glucose levels may serve as a key laboratory biomarker. Brain MRI demonstrates multiple and diverse intracranial lesions, with ring enhancement accompanied by ring diffusion restriction potentially representing relatively specific imaging features. mNGS of CSF may prove valuable for early diagnosis. Standardized antifungal therapy, particularly early intrathecal administration, may be critical to reducing mortality.

## Introduction

*Malassezia* species comprise a group of lipophilic yeasts that commonly colonize the skin surface of normal individuals. It is an opportunistic pathogen and is associated with a variety of dermatological conditions. While generally harmless in healthy individuals, these fungi pose significant threats to immunocompromised patients, where they can breach the skin barrier and cause systemic infections (Chang and Stein, 2024). Recent epidemiological data indicate that *Malassezia* infections have increased in recent years due to the increased use of immunosuppressive therapies. And the incidence of invasive infections, including life-threatening conditions such as catheter-related bloodstream infections, necrotizing pneumonia, and necrotizing enteritis, is increasing, with mortality rates exceeding 50 percent (Carreira et al., 2018; Saunte et al., 2020). Of particular concern is *Malassezia restricta*, which presents distinct clinical diagnostic and therapeutic challenges due to its lipid dependence, atypical clinical presentation, and challenging *in vitro* culture (Arendrup et al., 2014; Forster et al., 2022). Reviews of domestic and international literature revealed that there are extremely rare central nervous system (CNS) infections caused by *Malassezia restricta*. This study presents five novel cases of *Malassezia restricta*-induced meningoencephalitis. Comprehensive analysis incorporating clinical manifestations, metagenomic next-generation sequencing (mNGS) of cerebrospinal fluid (CSF) results, and multimodal neuroimaging evidence, we report for the first time that intracranial *Malassezia restricta* infection can lead to parenchymal abscess formation, exhibiting ring-enhancing lesions with central diffusion restriction‐a distinctive imaging pattern described as “ring enhancement with restricted diffusion”, which represents the first documentation of intracranial abscess formation by this pathogen. Additionally, we developed an effective treatment protocol that combines early intravenous and intrathecal antifungal therapy. Our results demonstrate that prompt diagnosis and tailored management can significantly improve survival outcomes in these challenging cases.

## Methods

This clinical study analyzed real-world data from patients diagnosed with M. restricta-induced meningoencephalitis, who were admitted to the Affiliated Aerospace Hospital of Zunyi Medical University before December 31, 2024. The research encompassed comprehensive patient data collection, including: 1) Demographic information. 2) Clinical manifestations. 3) Diagnostic examination results including brain magnetic resonance imaging (MRI), electroencephalogram (EEG), and CSF results. 4) mNGS findings. The study protocol was approved by the ethics review committee of the hospital and informed consent was obtained from all participants or their legal guardians. Data supporting the findings of this study are available from the corresponding author upon reasonable request.

Meningoencephalitis was defined using the following criteria (Venkatesan et al., 2013): Major Criterion (required): Patients presenting to medical attention with altered mental status (defined as decreased or altered level of consciousness, lethargy or personality change) lasting ≥24 h with no alternative cause identified. Minor Criteria (2 required for possible encephalitis; ≥3 required for probable or confirmed encephalitis): (1) Documented fever ≥38°Cwithin the 72 h before or after presentation. (2) Generalized or partial seizures not fully attributable to a preexisting seizure disorder. (3) New onset of focal neurologic findings. (4) CSF WBC count ≥5/cubic mm. (5) Abnormality of brain parenchyma on neuroimaging suggestive of encephalitis that is either new from prior studies or appears acute in onset. (6) Abnormality on electroencephalography that is consistent with encephalitis and not attributable to another cause. (7) The CSF of mNGS suggested *Malassezia restricta* amplification.

## Results

### Patient 1

**Figure 1:**
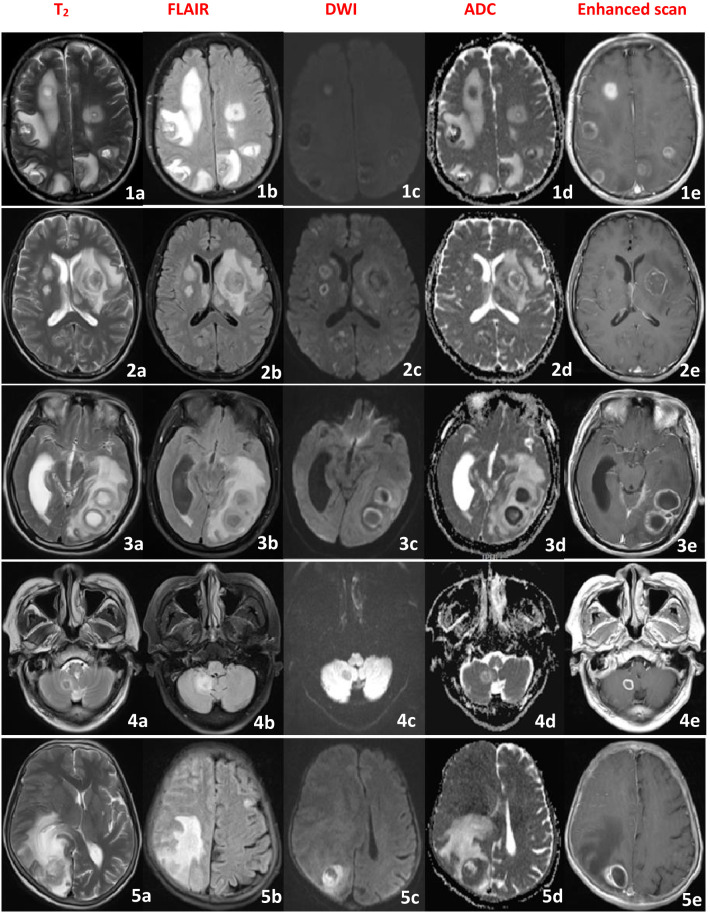
Brain MRI image of the patient (1-5), showing multiple intracranial circular lesions, High signal in the T2 and FLAIR sequence, low signal in the DWI and ADC in the center of the lesion, high signal around the lesion, and irregular circular enhancement in the enhanced scan.

A 68-year-old male, on October 17, 2022, presented with headache, fever, and decreased consciousness. Initially, he developed a diffuse, persistent throbbing pain in his forehead following a cold, accompanied by vomiting of his stomach contents. Oral analgesics were ineffective in relieving the headache. He also experienced severe-grade fever with temperatures fluctuating between 39-41°C. Upon admission to a local hospital, he was diagnosed with “upper respiratory tract infection” and treated with cefazolin for anti-infection, ibuprofen for pain relief, and antipyretic therapy. Despite these interventions, his headaches persisted and his fever continued. His condition progressively worsened, with psychotic symptoms such as incoherent speech and agitation. After two days of continuous treatment, the patient developed impaired consciousness, which progressed to a comatose state, prompting transfer to our hospital for further management. According to family members, he had a history of “pulmonary tuberculosis” ten years prior, which had been successfully treated. Physical examination revealed: temperature 40.7°C, pulse 138 bpm, respiratory rate 33 breaths/min, oxygen saturation 78%, blood pressure 132/67 mmHg. The patient was in a shallow coma, with reduced respiratory sounds in both lungs and neck stiffness (two-finger rigidity). Kernig’s sign was positive. Immediate interventions upon admission include endotracheal intubation with mechanical ventilation, continuous oxygen therapy, cardiac monitoring, and treatment with ceftazidime for infection control and ibuprofen for pain relief and fever reduction. Rapid CSF tests demonstrated a yellow appearance, significantly elevated intracranial pressure, increased CSF white blood cell (WBC) count and protein levels, mildly decreased chloride, and markedly reduced glucose (**Table 1**). Blood tests showed WBC of 41.29×10^9/L, neutrophils 92.7%, lymphocytes 2.5%, erythrocyte sedimentation rate (ESR) 67 mm/h, and C-reactive protein (CRP) 158.4 μg/ml. Video EEG revealed diffuse slow waves in both hemispheres of the brain. Brain MRI identified multiple rounded nodular lesions in both hemispheres, with ring-like enhancement on contrast scans (**Figure 1**, patient 1a-e). Chest computed tomography (CT) indicated bilateral pulmonary infection. CSF and serum tests for autoimmune encephalitis (AE) antibodies, including myelin oligodendrocyte glycoprotein (MOG), glial fibrillary acidic protein (GFAP), and aquaporin-4 (AQP4) antibodies, serum tuberculosis (TB) antibodies, CSF acid-fast staining, and ink staining for Cryptococcus were all negative. No pathogenic microorganisms were detected in blood or CSF cultures. The mNGS of the CSF identified a significant amplification of *Malassezia restricta*. The diagnosis was severe meningoencephalitis caused by *Malassezia restricta* infection. The treatment regimen was promptly adjusted to include intravenous and intrathecal amphotericin B for antifungal therapy, intravenous dexamethasone for inflammatory response mitigation, continued ceftazidime for infection control, and ibuprofen for fever and pain relief. After one week of treatment, the patient gradually regained consciousness, with body temperature dropping to 38°C and improved psychiatric symptoms. After two weeks of continuous therapy, the endotracheal tube was removed and the patient was successfully weaned off the ventilator. Unfortunately, on the 25th day of treatment, the patient’s condition suddenly worsened, with recurrent comas and seizures. The family chose to discontinue treatment and the patient was discharged. A follow-up call revealed that the patient died approximately two months after discharge due to the deterioration of the disease.

**Table 1:** Cerebrospinal fluid laboratory results for patients.

CSF	Intracranial pressure (mmH_2_O)	WBC (×10^6^/L)	Nucleated cell (×10^6^/L)	Glucose (mmol/L)	Chloride (mmol/L)	Protein (mg/L)
Patient 1						
No treatment	>400	450	170	1.03	116.8	2190
Post-treatment	180	68	40	2.67	122.4	757
Patient 2						
No treatment	>400	300	120	1.17	107.2	683
Post-treatment	140	12	8	4.18	122.6	482
Patient 3						
No treatment	370	10	8	2.1	123.6	675
Post-treatment	150	5	2	4.67	120.4	328
Patient 4						
No treatment	340	120	68	1.27	125.8	1940
Post-treatment	190	14	8	3.12	128.6	458
Patient 5						
No treatment	>400	690	320	0.79	118.2	1864
Post-treatment	200	24	7	2.8	122.7	657

CSF, cerebrospinal fluid; WBC, white blood cell.

### Patient 2

A 70-year-old female, on April 8, 2023, presented with headaches, abnormal mental behavior, and tics. Initially, she experienced persistent throbbing headaches accompanied by nausea and vomiting, and was unresponsive to oral analgesics. Subsequently, she developed disturbances in mental behavior characterized by incoherent speech and limbic movements. She sought treatment at a local hospital where she was diagnosed with “viral encephalitis and epilepsy.” Her initial treatment regimen included acyclovir for antiviral therapy, ibuprofen for pain relief, and valproate for seizure control. Despite three days of continuous treatment, her headaches showed no significant improvement and her symptoms of incoherent speech and repetitive movements persisted without relief, prompting her to be transferred to our hospital. Her medical history reveals a long-standing involvement in poultry. On physical examination, she exhibited a delirious state, nuchal rigidity (two-finger breadth), and a positive Kernig’s sign. Further investigation included video EEG, which demonstrated diffuse low-wave with spike and sharp-wave discharges. Brain MRI revealed multiple round nodular lesions in both cerebral hemispheres, showing ring-like enhancement on contrast imaging (**Figure 1**, patient 2a-e).CSF tests indicated a colorless and transparent appearance, significantly elevated intracranial pressure, increased CSF white cell count, markedly elevated CSF protein, significantly reduced glucose levels, and mildly reduced chloride levels (**Table 1**). Blood tests, including white blood count, ESR, and CRP, were within normal limits. CSF and serum tests for AE antibodies (including MOG, GFAP, and AQP4 antibodies) were negative. Serum TB antibody tests were also negative, as were CSF acid-fast staining and ink staining for Cryptococcus. No pathogenic microorganisms were detected in successive serum and CSF cultures. However, the mNGS of the CSF identified a significant amplification of *Malassezia restricta*. Based on these findings, a definitive diagnosis of severe meningoencephalitis due to *Malassezia restricta* infection was established. The treatment regimen was promptly adjusted to include intravenous and intrathecal amphotericin B antifungal therapy. During administration, patients developed infusion-related reactions, including rapid breathing and rash, requiring immediate discontinuation. Symptoms improved after treatment with chlorphenamine and calcium gluconate. Fluconazole was subsequently added to the antifungal regimen. Additional therapies included mannitol for intracranial pressure reduction, valproate for seizure control, and ibuprofen for pain relief. After ten days of continuous treatment, the patient’s headaches and seizures improved significantly and her psychiatric symptoms eased. After forty-four days of continuous treatment, she showed no signs of headache, seizures, or fever, was perfectly conscious, and exhibited no incoherent speech. The follow-up EEG revealed a marked reduction in moderate wave activity compared to baseline, with no spikes or sharp wave discharges noted. Repeated CSF results demonstrated significant improvements across all parameters. The patient was released from the hospital in stable condition.

### Patient 3

A 56-year-old male, on November 20, 2023, presented with impaired consciousness and seizures. He was immediately admitted to a local hospital after suffering a sudden loss of consciousness, accompanied by convulsions of his limbs while resting. The brain CT scan showed no abnormalities, and the diagnosis was “acute cerebral infarction. “Intravenous thrombolysis with alteplase was administered. Despite thrombolysis, the patient remained comatose with constant involuntary limb movements. Follow-up CT angiography (CTA) of the head and neck revealed no vascular stenosis. His condition deteriorated, characterized by generalized tonic-clonic seizures, prompting his transfer to our hospital due to the critical nature of his condition. The patient had a lengthy history of bedridden immobility. On physical examination, the patient was in a light coma with spontaneous limb movements and increased muscle tone in all extremities. Signs of meningeal irritation were negative and pathological reflexes were absent. Brain MRI findings demonstrated large patchy lesions in the left temporal-occipital lobe, contrast-enhanced scans revealed ring-like enhancement (**Figure 1**, patient 3a-e). the video EEG shows diffuse low waves with paroxysmal spikes and sharp waves in the left hemisphere. CSF results indicated clear and colorless fluid, elevated intracranial pressure, normal of cell count, mildly increased protein levels, and normal chloride and glucose levels (**Table 1**). Laboratory tests revealed an ESR of 48 mm/h and CRP of 67.4 μg/ml. Testing for AE antibodies (including MOG, GFAP, and AQP4) in both CSF and serum was negative. Serum TB antibodies were also negative, as were CSF acid-fast staining and ink-staining of prokaryotes. Serial cultures of serum and CSF did not reveal any pathogenic microbial growth. *Malassezia restricta* was identified in the mNGS of CSF. Definitive diagnosis: *Malassezia restricta*-induced meningoencephalitis. Treatment includes intravenous amphotericin B in combination with antifungal therapy, dexamethasone for inflammation reduction, mannitol for intracranial pressure control, and valproate for seizure management. After seven days of treatment, the seizures subsided and the patient regained consciousness. By the tenth day of treatment, the patient was fully alert and had no recurrent seizures. Follow-up EEG demonstrated reduced low waves without spikes or sharp wave discharges. Repeated CSF tests confirmed the normalization of all parameters. The patient was released from the hospital in an improved condition.

### Patient 4

A 76-year-old male, on June 22, 2024, presented with complaints of headache and fever. Initially, the patient experienced a persistent, throbbing headache localized to the top of the head, accompanied by nausea, vomiting, and unresponsiveness to oral analgesics. Subsequently, he developed a fever with a temperature fluctuating between 38°C~40°C.He was admitted to a local hospital and diagnosed with “meningitis.” Treatment includes anti-viral treatment with acyclovir and ibuprofen for pain relief and fever reduction. Despite three consecutive days of treatment, the patient’s headache persisted and the fever continued without improvement. The patient was then transferred to our hospital for further diagnostic evaluation. No significant past medical history was reported. Physical examination revealed clear consciousness but lethargy, normal muscle strength in all limbs, negative signs of meningeal irritation and no pathological reflexes. Brain MRI demonstrated a circular lesion in the right cerebellar hemisphere with ring enhancement (**Figure 1**, patient 4a-e).The video EEG shows diffuse low waves predominantly in the right hemisphere. CSF results revealed a colorless and transparent appearance, elevated intracranial pressure, slightly increased WBC, markedly decreased glucose levels compared to serum glucose, normal chloride levels, and significantly elevated protein levels (**Table 1**). Blood tests showed WBC 8.64×10^9/L, N 62.7%, L 37.3%; ESR 48 mm/h, CRP 87 μg/ml. CSF and serum AE antibodies, MOG, GFAP and AQP4 antibodies were all negative. Serum TB antibodies were also negative, as were CSF acid-fast staining and cytomegalovirus ink staining. No pathogenic microorganisms were detected in serum or CSF cultures, while mNGS of CSF identified amplification of *Malassezia restricta*. The diagnosis was meningoencephalitis caused by *Malassezia restricta* infection. Treatment consists of intravenous amphotericin B in combination with intrathecal amphotericin B for antifungal therapy, mannitol for intracranial pressure reduction, and ibuprofen for analgesic and fever management. After five days of continuous treatment, the patient’s headaches improved. After two weeks of treatment, his temperature gradually normalized. After six weeks of treatment, the patient became asymptomatic, without headaches or fever. Repeated EEG showed a marked reduction in low-wave activity, and repeated CSF tests demonstrated reduced intracranial pressure, significantly reduced protein levels, and normalized cell counts. The patient was released from the hospital in an improved condition.

### Patient 5

A 73-year-old female, on February 12, 2025, presented with complaints of headache and decreased consciousness. Initially, she reported a persistent, throbbing headache localized on the top of her head, accompanied by nausea, vomiting, and nonresponsiveness to oral analgesics. She was admitted to a local hospital and diagnosed with “upper respiratory tract infection,” receiving ribavirin antiviral therapy. However, after three days of treatment, her condition deteriorated, manifesting in loss of consciousness, seizures, and necessitating transfer to our hospital for further evaluation. The patient had a ten-year history of rheumatoid arthritis managed with intermittent oral prednisone therapy. On physical examination, she was in a light coma with neck stiffness (three fingers), and a positive Kernig sign. Video EEG demonstrated diffuse low waves, paroxysmal sharp waves, and right hemisphere spikes. Brain MRI revealed large flake-like circular lesions in the right hemisphere, with ring enhancement on contrast-enhanced imaging (**Figure 1**, patient 5a-e). A chest CT revealed a bilateral lung infection. CSF results revealed a yellowish appearance, significantly elevated intracranial pressure, increased white cell count, markedly reduced glucose and chloride levels, and elevated protein concentration compared to serum (**Table 1**). Blood tests indicated WBC 38.07×10^9/L, N 88.7%, L 11.3%; ESR 146 mm/h, CRP 254.9 μg/ml. CSF and serum AE antibodies including MOG, GFAP and AQP4 were all negative. Serum TB antibodies were also negative, as were CSF acid-fast staining and cytomegalovirus ink staining. Continuous cultures of serum and CSF showed no growth of pathogenic microorganisms, while mNGS of CSF identified significant expansion of *Malassezia restricta*. Based on these findings, the diagnosis was severe meningoencephalitis caused by *Malassezia restricta* infection. Treatment includes intravenous amphotericin B for antifungal therapy, dexamethasone for inflammatory response reduction, ceftazidime for infection control, mannitol for intracranial pressure reduction, sodium valproate for seizure control, and ibuprofen for pain relief. After ten days of continuous treatment, the patient’s consciousness improved from a light coma to lethargy, with intermittent seizures and fever. After six weeks of intensive treatment, her consciousness progressed from lethargy to lucidity, the headaches resolved and the seizures were gradually brought under control. Repeated EEG showed reduced low-wave activity and reduced scattered bifurcations and sharp waves, while repeated CSF tests demonstrated overall improvements in all parameters. The patient was discharged with marked clinical improvement.

## Discussion

This study presents a detailed analysis of real-world clinical case data obtained from a tertiary level A hospital in Zunyi, Guizhou, China. Data were collected on five cases of meningoencephalitis caused by *Malassezia restricta* infection. The clinical characteristics of the patients, laboratory test results and imaging findings were comprehensively analyzed. Our findings indicate that CNS infections due to *Malassezia restricta* typically present as acute-onset, critical conditions with rapid deterioration. Headache is the dominant clinical symptom, while altered consciousness may serve as an essential indicator of severe illness. CSF testing typically lacks specific variants. However, a significant increase in intracranial pressure and a significant decrease in CSF glucose levels may act as key laboratory biomarkers. Brain MRI demonstrates multiple and diverse intracranial lesions, with ring enhancement accompanied by ring diffusion restriction potentially representing relatively specific imaging features. Given the challenges associated with *in-vitro* culture, mNGS of CSF proved valuable for early diagnosis.

In 1981, Redline and Dahms documented the first case of sepsis in preterm infants receiving lipid infusions due to *Malassezia* infection, which affected multiple organs, including the heart, lungs, and blood vessels (Redline and Dahms, 1981). In 1989, a case was reported involving extensive dissemination to additional organs, such as the kidneys, pancreas, colon, adrenal glands, liver, spleen, brain, and meninges, beyond the lungs and heart (Shek et al., 1989). Subsequent reports have described cases of *Malassezia* infections in children, with studies indicating that preterm birth, prolonged neonatal intensive care unit stays, and intravenous lipid administration are the predominant risk factors for such infections (Rosales et al., 2004; Kucinskiene et al., 2014; Zhang et al., 2023). However, CNS infections caused by *Malassezia* remain relatively rare. Cases reported in children are predominantly secondary to infections originating elsewhere in the body, and CNS infections specifically attributed to *Malassezia restricta* remain a blank. Consequently, the precise mechanisms underlying CNS infection caused by *Malassezia restricta* remain unknown.

Neuroinflammation is a hallmark of central nervous system diseases, resulting in neuronal damage and degeneration. The main mechanisms include microglial activation (Muzio et al., 2021), astrocyte activation (Singh, 2022), abnormal expression of cytokines (Jeon and Kim, 2016; Ermakov et al., 2023), blood-brain barrier dysfunction (Takata et al., 2021), and oxidative stress, etc (Solleiro-Villavicencio and Rivas-Arancibia, 2018). Among them, cytokines play a key role in causing inflammation in neural tissues. Studies have shown that both *in vitro* and *in vivo* experiments can reveal the ability of *Malassezia* to induce the production of various cytokines (Buentke et al., 2001; Watanabe et al., 2001; Corzo-León et al., 2020; Wolf et al., 2021).These findings suggest that *Malassezia* plays a role in the production of inflammatory cytokines associated with neurological diseases, potentially influencing the prognosis and severity of these diseases.

Regarding the pathogenic mechanism of *Malassezia restricta*, studies have shown that host inheritance plays a key role in human invasive fungal diseases, and the genetic polymorphisms of certain genes increase the susceptibility of *Malassezia restricta* to fungal infections (Pana et al., 2014; Naik et al., 2021). Animal model discovery: Innate and adaptive immune mechanisms play a significant role in central nervous system infections caused by *Malassezia restricta* (Naik et al., 2025). It is well known that caspase recruiting domain protein 9 (CARD9) is an important component of the innate immune system, which recruits neutrophils from the central nervous system. It plays a key role in controlling fungal invasion (Drummond et al., 2014; Gilbert et al., 2014). In addition, CARD9 can activate CNS microglia to induce the release of pro-inflammatory cytokine IL-17 against *Malassezia restricta* (Sparber et al., 2019). Meanwhile, *Malassezia restricta* has developed multiple mechanisms to evade or resist these immune responses, such as the formation of thick cell walls (Billamboz and Jawhara, 2023), biofilm formation, etc (Hobi et al., 2022). These strategies enable *Malassezia restricta* to persist and settle in different host niches, making patients with weakened immune systems or immunodeficiency diseases more susceptible to *Malassezia restricta* infection (Gaitanis et al., 2012). In the central nervous system, the blood-brain barrier (BBB) provides crucial resistance against the entry of pathogens (Deane et al., 2003; Zhao et al., 2024). Another factor leading to *Malassezia restricta* infection is the disruption of the skin barrier. The disruption of the skin barrier can change the composition of the microbiota, causing disorders in the brain-gut axis pathway and resulting in the disruption of the blood-brain barrier, ultimately triggering inflammatory and immune responses in the nervous system (Rutsch et al., 2020).

Furthermore, *Malassezia restricta* has a highly conserved fungal zinc transport system (Park et al., 2017). In yeast cells treated with Zinc pyrithione(ZPT), the homologous gene expression of vacuolar zinc transporter protein in Saccharomyces cerevisiae was significantly upregulated, indicating an increase in zinc levels in fungal cells (Reeder et al., 2011). The results of transcriptomic analysis showed that ZPT treatment could up-regulate the genes related to the biosynthesis of the Fe-S cluster in *Malassezia restricta* (Roques et al., 2006). Studies have shown that there is a significant increase in zinc accumulation in ZPT-treated *Malassezia restricta* cells, which leads to a down-regulation of SOD2 regulation, a reduction in heme synthesis, and a decrease in the synthesis of the Fe-S cluster (Galiazzo and Labbe-Bois, 1993; Brown et al., 2004). It was observed in Saccharomyces cerevisiae that the transcription of SOD2 was significantly reduced, and the reduction in heme synthesis mainly occurred in the mitochondria (Carraway et al., 2006; Mühlenhoff et al., 2015). Studies have shown that excessive accumulation of zinc in cells leads to the accumulation of reactive oxygen species in organelles (Gazaryan et al., 2002; Dineley et al., 2003). The accumulation of reactive oxygen species reduces ATP production, interferes with glycolysis, the tricarboxylic acid cycle and the electron transport chain, and ultimately leads to mitochondrial dysfunction (Lemire et al., 2008; Park et al., 2018). Therefore, it is speculated that *Malassezia restricta* infection is closely related to mitochondrial dysfunction.

Our comprehensive analysis of the clinical characteristics of the five rare cases revealed that most of the patients were older at the onset of the disease, with four patients aged over 60 years and three over 70 years. Additionally, three patients had a history of immune dysfunction (including tuberculosis, long-term bed rest, and oral hormone use). Notably, one of the patients not had a documented history of immunodeficiency (**Table 2**). Previous literature has reported that most CNS infections caused by *Malassezia* occur in immunocompromised individuals (Guillot and Bond, 2020). However, our study identified cases in patients with normal immune function, raising the question of whether the pathogenicity of *Malassezia restricta* may be influenced by microflora imbalance or patient genetic susceptibility. Therefore, constructing *in vitro* models of the human blood-brain barrier and exploring the association between lipid metabolism and barrier disruption using metabolomics could be a promising focus for future research.

**Table 2:** Clinical characterization of patients.

Clinical characterizes	Patient 1	Patient 2	Patient 3	Patient 4	Patient 5
Six	Male	Female	Male	Male	Female
Age(years)	68	70	56	76	73
Headache	+	+	+	+	+
Consciousness disorders	+	+	+	–	+
Psychiatric symptoms	–	+	–	+	–
Seizures	+	–	+	–	+
Fever	+	+	+	–	+
Brain MRI	abnormal	abnormal	abnormal	abnormal	abnormal
ECG	abnormal	abnormal	abnormal	abnormal	abnormal

MRI, magnetic resonance imaging; ECG, electroencephalogram; CSF, cerebrospinal fluid.

In all patients with acute onset, headache is the dominant clinical symptom, cognitive impairment, psychiatric symptoms, and seizures may also occur. Notably, these clinical manifestations lack specificity. Importantly, we observed that patients who exhibited impaired awareness tended to have more severe disease deterioration. Brain MRI in all patients revealed intracranial lesions, which were either solitary or multiple, involving both the brain parenchyma and meninges. These findings do not differ significantly from those typically associated with common bacterial infections. A comparative analysis of craniofacial MRI results demonstrated pus-like changes in the cerebral parenchyma on plain scans, during diffusion-weighted imaging (DWI),the lesions around exhibited restricted diffusion. Furthermore, and show the varying degrees of loop enhancement on augmented scans. While most previous studies have reported nonspecific findings such as meningeal enhancement or cerebral oedema, our case uniquely demonstrates ring-enhanced cerebral parenchymal lesions around with restricted diffusion the DWI sequence. This finding aligns closely with the “fungal microabscess” structure described in histopathological studies (Gaitanis et al., 2012). Histopathological confirmation has shown that the central region of the microbe exhibits a ring-shaped diffusivity restriction due to the accumulation of fungal hyphae, while the surrounding region represents an inflammatory cell percolation zone resulting in a ring-shaped enhancement (Arendrup et al., 2014; Masouris et al., 2025).Our findings suggest that *in vivo* validation of the pathological evolution of CNS infection by *Malassezia restricta* provides critical imaging evidence for elucidating the invasion mechanism. Consequently, we propose for the first time that the “Ring enhancement accompanied by ring diffusion restriction sign” could serve as a key imaging biomarker for the early diagnosis of *Malassezia restricta*-related infections. However, given the limited number of cases in our study, further large-scale investigations are warranted for definitive confirmation.

CSF results range from mostly normal to biochemical changes characteristic of tuberculous meningitis. However, significant increases in intracranial pressure and marked decreases in CSF glucose were observed in the majority of patients. Based on patient prognosis, we find that low CSF glucose appears to be a critical indicator of poor outcomes. Previous studies have demonstrated that traditional CSF culture has an exceptionally low positive rate for the detection of *Malassezia*, and that distinguishing *Malassezia* from skin-colonizing bacteria is challenging. Given the rarity of *Malassezia restricta* infections, the sensitivity and specificity of molecular detection techniques such as mNGS still require further validation (Chang et al., 2020; Peng et al., 2024). However, due to the extreme difficulty of culturing *Malassezia in vitro* (Alameer et al., 2025), we opted for CSF mNGS, which significantly improved the detection rate. Therefore, we believe that this technique facilitates early diagnosis. In terms of treatment, given the lack of consensus or guideline recommendations, we mention that other therapeutic strategies for fungal meningitis and the administration of standardized intravenous antifungal therapy in combination with intrathecal injections have significantly improved patient outcomes. Although international guidelines recommend amphotericin B liposomes as the primary antifungal regimen, there remains insufficient evidence regarding the necessity of combined intrathecal administration and the optimal duration of antifungal therapy (Redline and Dahms, 1981; Zhang et al., 2023).Therefore, Further studies are needed to investigate the efficacy and safety of combination therapy.

However, our study has certain limitations. First, this is a single-center clinical case study, which may introduce selection bias in the collected data. Second, the patient did not undergo a brain biopsy puncture. Thus, we lack a gold standard for definitive pathological confirmation. Moreover, given the relatively short duration of this study, extended follow-up is essential to assess the long-term prognosis of neurological function in patients. Nonetheless, a large sample controlled study within a single center is challenging due to the extremely limited number of cases of severe meningoencephalitis caused by *Malassezia restricta* infection in our region and nationally. We anticipate further collaborations with different medical institutions to address these limitations. Despite these challenges, real-world registry data remains particularly valuable, as randomized controlled trials are often impractical in the context of rare diseases. Such data can provide critical clinical reference points and support subsequent mechanistic investigations.

## Conclusion

The severe meningoencephalitis caused by Malassezia restricta is an exceptionally severe CNS infectious disease characterized by clinical rarity, misdiagnosis, and fatality rate. The disease is usually acute in onset, with rapid deterioration of critical conditions. Headache is the dominant symptom, while decreased consciousness may serve as an essential indicator of severe illness. CSF usually shows a significant increase in intracranial pressure, and a significant decrease in CSF glucose levels may serve as a key laboratory biomarker. Brain MRI demonstrates multiple and diverse intracranial lesions, with ring enhancement accompanied by ring diffusion restriction potentially representing relatively specific imaging features. mNGS of CSF may prove valuable for early diagnosis. Further confirmation is anticipated from multicenter, large-sample randomized controlled trials.

## Data Availability

The datasets presented in this study can be found in online repositories. The names of the repository/repositories and accession number(s) can be found in the article/supplementary material.
